# Quality of life after upper third molar removal: A prospective longitudinal study

**DOI:** 10.4317/medoral.21781

**Published:** 2017-10-21

**Authors:** Vanesa Avellaneda-Gimeno, Rui Figueiredo, Eduard Valmaseda-Castellón

**Affiliations:** 1 DDS. Fellow of the Master degree program of Oral Surgery and Implantology. School of Dentistry of the University of Barcelona (Spain); 2 DDS, MS, PhD. Associate Professor of Oral Surgery. Coordinator of the Master degree program in Oral Surgery and Implantology. School of Dentistry of the University of Barcelona (Spain). Researcher of tbe IDIBELL Institute, Barcelona (Spain); 3 DDS, MS, PhD. Professor of Oral Surgery. Director of the Master degree program in Oral Surgery and Implantology. School of Dentistry of the University of Barcelona (Spain). Researcher of tbe IDIBELL Institute, Barcelona (Spain)

## Abstract

**Background:**

Third molar extraction is a very common procedure in Dentistry. The aim of this study was to evaluate the quality of life (QoL) and satisfaction of patients undergoing extraction of an upper third molar under local anesthesia. A second objective was to describe the evolution of self-reported pain measured in a visual analogue scale (VAS) in the 7 days after surgery and its relationship with pre- and intraoperative factors.

**Material and Methods:**

A prospective longitudinal cohort study was made. Fifty-five patients received a questionnaire assessing social and working isolation, eating and speaking ability, diet modifications, sleep impairment, physical appearance, discomfort at suture removal and overall satisfaction. Pain was registered daily on a VAS scale. A descriptive and bivariate analysis of the data was performed.

**Results:**

Forty-seven patients were included. Pain decreased lineally across the 7 days, and relief was significant between days 2 and 3. Intraoperative complications were significantly associated with pain. The complication that showed the highest pain score was the tuberosity fracture.

**Conclusions:**

Upper third molar removal significantly affects the patient’s quality of life, particularly during the first 2 days after extraction.

** Key words:**Quality of life, upper third molar, extraction, complications.

## Introduction

The term quality of life (QoL) describes a multidimensional concept concerning the ability of the patients to carry out their daily activities (1-3).

QOL is a concept difficult to be assessed considering that the result might have differences depending on individual perception. However, the questionnaires to assess QoL are designed to measure the quality, the effectiveness and the efficiency of the treatment methods as well as physical, psychological and social consequences for patients with different health states (2).

Most people require the extraction of the third molar at some time in life mostly due to pain, tooth decay or periodontal disease. Therefore, third molar extraction is still one of the most frequent interventions in oral surgery (2-4). Pericoronitis is the most frequent indication for the extraction of third molars (5). Furthermore; there are other indications, such as infection, restorative reasons or caries. Prophylactic indication or prevention of crowding are more controversial.

As in any surgery, the extraction of the upper third molar causes tissue damage and has an impact both at local and systemic level that deteriorates the QoL of the patient (6,7). The upper third molar extraction can require raising a flap, removing bone or even sectioning the tooth. Different regimes of postoperative medications have been described. Indeed, patients undergoing surgical extraction of third molars suffer alterations in their daily routine, mainly caused by pain and swelling (6,8,9). Although the number of studies evaluating the influence on the QoL of patients during the postoperative period after undergoing different treatments of oral surgery is growing (1,2,7,10), to date there is no information on the impact on QoL in the postoperative course after the surgical extraction of the upper third molars. Therefore patients and clinicians can only rely on clinical experience to predict this impact.

The main objective of this report was to measure the QoL for the first 7 days after the removal of a third upper molar, using a previously validated questionnaire (1,2).

The secondary objective was to measure by means of a validated questionnaire the degree of satisfaction of outpatients under-going extraction of the third upper molars (1,2), to assess the evolution of the postoperative pain during the first 7 days after the extraction and to describe the need for analgesic consumption.

## Material and Methods

Patients who had an appointment to extract an upper third molar at the Master of Oral Surgery and Orofacial Implantology of the University of Barcelona were recruited for the study.

Inclusion criteria were: patients older than 18 years old requiring an extraction of a single upper third molar. Exclusion criteria were: systemic diseases (ASA III or higher) that contraindicate surgery or impair wound healing, patients with antibiotic premedication or under pharmacological treatment that might interfere with wound healing, patients with contraindications of the extraction under local anesthesia, patients on chronic NSAID therapy, and patients unable to understand the visual analogue scales or the questions related to the QOL. If another tooth had to be extracted in the same appointment, the patient was excluded as well.

The study protocol was approved by the Institutional Review Board (Comitè Ètic d’Investigació Clínica) before recruitment of the patients. The guidelines of the Helsinki Declarations were considered and followed through all the study. Patients signed an informed consent for the participation in this study. All extractions were preformed from March 2015 to June 2015 at the Dental Hospital of the University of Barcelona by postgraduate students (first, second or third year) of the Master of Oral Surgery and Orofacial Implantology of the University of Barcelona. Data were collected by a single investigator who was not involved in the surgery.

The molars were extracted for prophylactic reasons, pericoronitis, or orthodontic reasons. In case it was required, a full-thickness flap was raised and bone removal was performed using a round carbide bur on a straight handpiece. Third molars were extracted using Pott elevators. If a flap was raised, the wound was closed with 3-0 silk sutures.

The instruction about postoperative medication was 600mg ibuprofen TID during 3-5 days. Antibiotics were prescribed when bone removal was required (amoxicillin 750 mg TID during 7 days).

The variables registered were: age, gender, side of the third molar, eruption status, bone retention, previous symptoms, bone re-moval, tooth sectioning, experience of the surgeon, and intraoperative complications such as mucosal tear or fracture of the tuberosity. For 7 days patients recorded the average pain on a 10 cm visual analogue scale (VAS) and the number of consumed analgesic or NSAID tablets.

The same day of the surgery, patients received the first questionnaire ( Table 1) and the visual analogue scales sheets and were instructed about how and when to fill them. Seven days after surgery suture were removed and the completed questionnaire with information about pain and postoperative quality of life was collected. At that time another questionnaire ( Table 2) was filled by the patient.

**Table 1:**
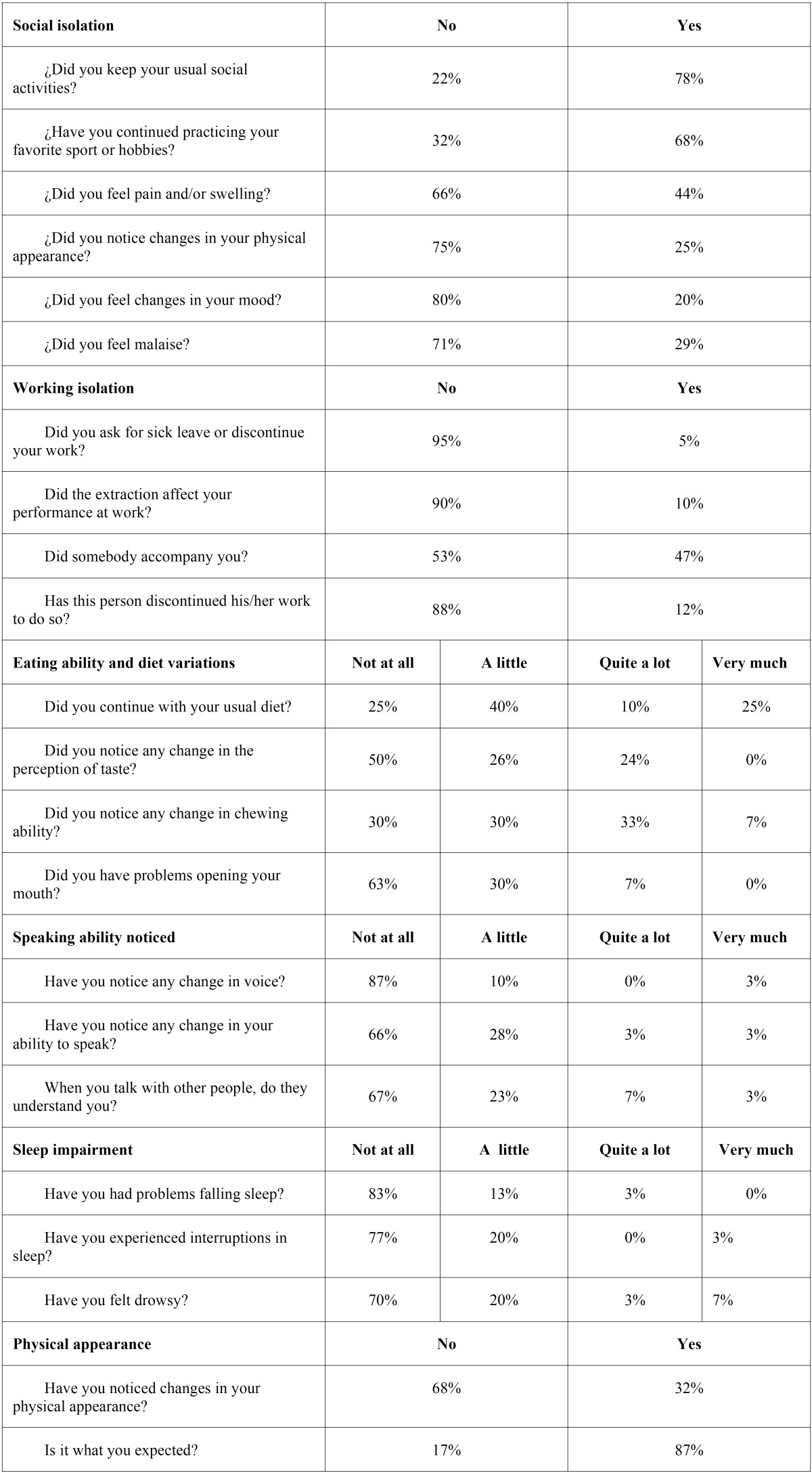
Questionnaire filled during the first postoperative week and returned at day 7 after surgery.

**Table 2:**
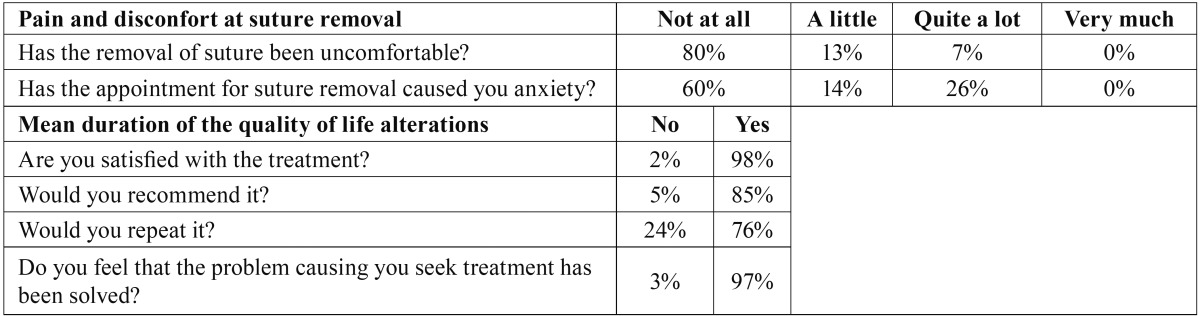
Questionnaire filled and returned after suture removal at day 7.

Data were processed using IBM SPSS version 22.0 (IBM Corp; Armonk, NY, USA).

T-tests were used to compare the duration of the changes (in days) in males and females. The duration of the changes for the eruption status, bone retention, presence of previous symptoms, bone removal, tooth sectioning, experience of the surgeon, and intraoperative complications were assessed with one-way ANOVA tests.

The association of pain with gender was measured by Pearson’s χ²-tests. The pain VAS scores were analyzed by analysis of variance (ANOVA) for repeated measures with the Greenhouse-Geisser correction if sphericity did not hold and post-hoc contrasts with the Bonferroni correction.

## Results

Questionnaires were delivered to 55 patients. Forty-seven were returned (30 females and 17 males). Five patients lost the questionnaire and 3 failed to fill it correctly and were excluded from the study. The mean age was 36 years, with a standard deviation (SD) of 9.3 years. All patients were Caucasian. Questionnaire results are shown on  Table 1 and  Table 2.  Table 3 displays the distribution of the registered variables.

**Table 3:**
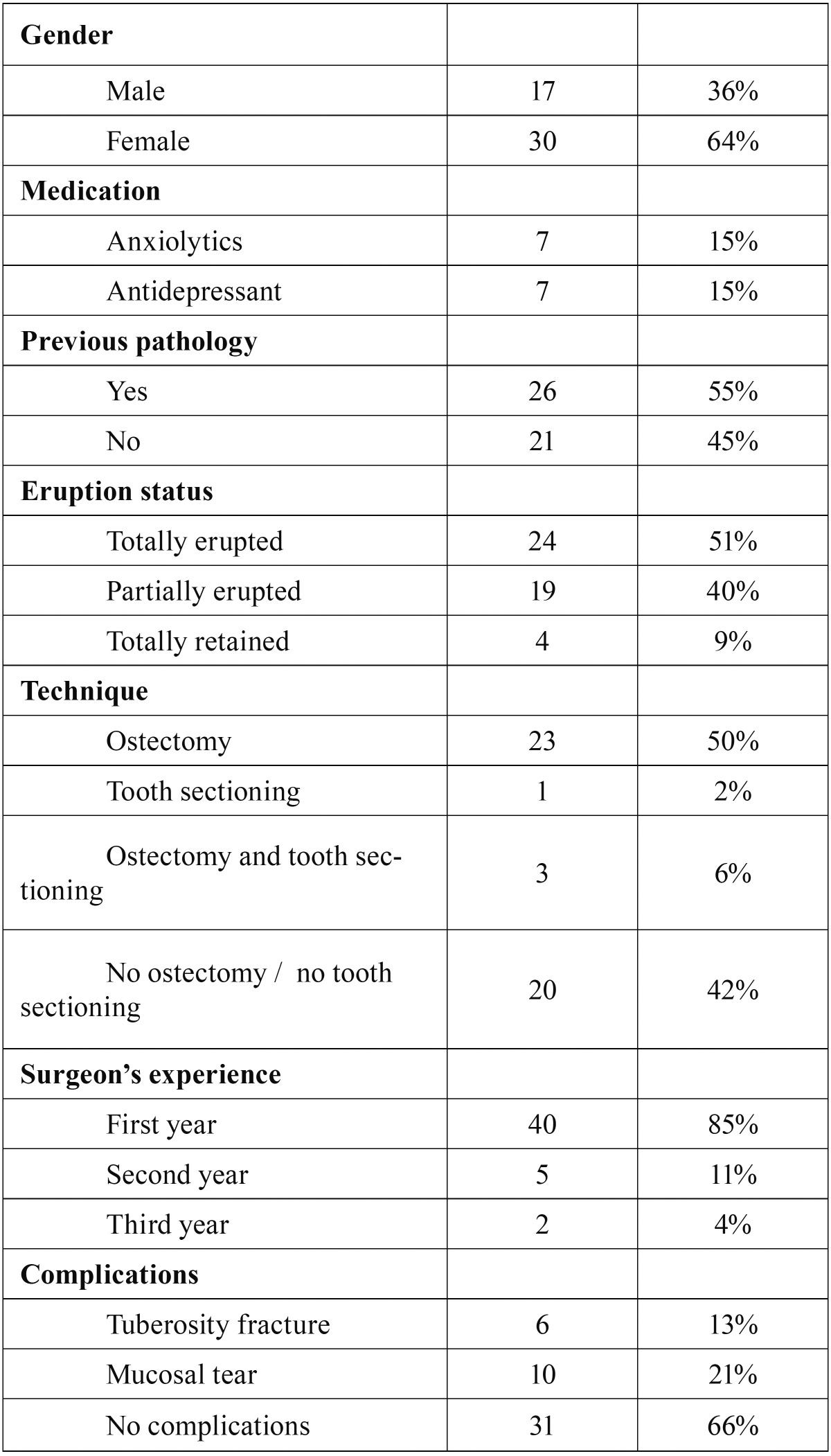
Demographic and operative data.

**Figure 1:**
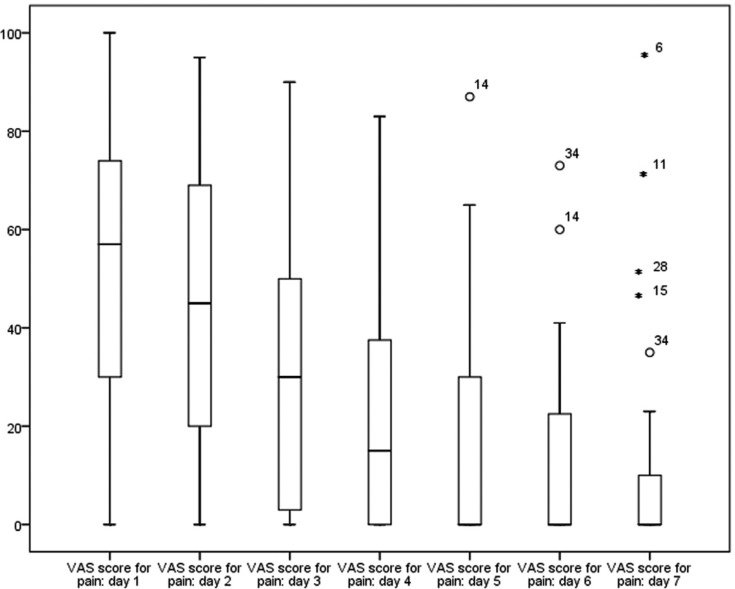
Boxplot VAS of pain during the postoperative period. The outliers show the patient’s identification number. The vertical axis represents VAS scores (from 0 to 100 mm). Circles represent outlier values. Asterisks represent extreme values. The graphic shows outliers with persistent pain on day 5 to 7, and an overall lineal reduction of pain.

The VAS score for pain across the 7 days showed a progressive reduction in pain intensity (F=66,121; degrees of freedom (df)=3,324; *P*<0.005), with a lineal pattern (F=234,286; df=1; *P*<0.005). On day 2 and day 3 this decrease in the VAS of pain was statistically significant (day 2 compared with day 3 and 4: *P*=0.035 and *P*=0.026 respectively) with a mean reduction of 16.3mm and 12.4mm respectively (Fig. 1).

**Figure 2:**
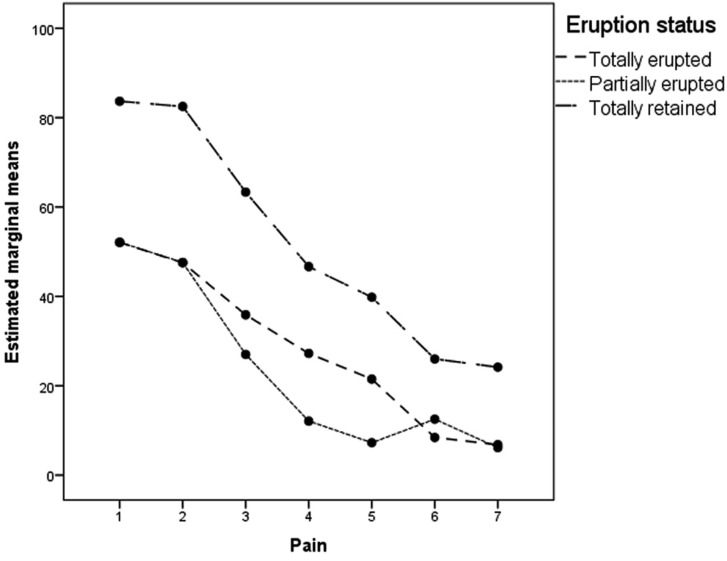
Pain score by eruption status. The horizontal axis represents the days after surgery.

Pain differences by gender were not significant, and the pattern of decline was similar for men and women (F=2.409; df=3.324; *P*=0.071). Pain scores were not significantly lower when neither bone removal nor tooth sectioning was performed. (F=1,642; df=9,972; *P*=0.120). Patients who had total retention of the upper third molar had higher VAS scores than those with partially or totally erupted molars (Fig. 2), but the difference was not significant (F=2,975; df=3 *P*=0.063).

**Figure 3:**
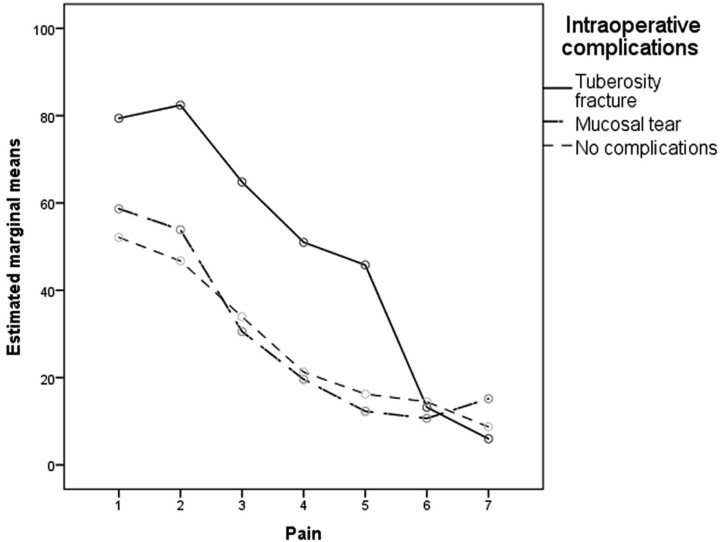
Pain score by complications. The horizontal axis represents the days after surgery.

There were no significant differences in pain between molars with and without pre-existing symptoms. (F=0.619; df=3.324; *P*=0.714). However, patients who had intraoperative complications had significant higher VAS scores than patients without complications (F=3.567; df= 6.648; *P*=0.004). The complication that showed the highest pain score was the tuberosity fracture (Fig. 3).

The average consumption of NSAID tablets was 1.2 per day. This intake was concentrated in the first 2 days (2.1 per day in the first 2 days). From the day 3 there was a 23% reduction of the inake of of analgesics, and from the day 4 a 50 % reduction.

## Discussion

A limitation of the present study is that data are subjective, based only on the patient perception. However, this is always an issue in studies of QoL (11,12). Another limitation of the study is that the questionnaire only collected information corresponding to the first 7 days after surgery; consequently, only the short-term evolution of the changes in QoL could be assessed. Therefore, possible long-term complications could not be evaluated, which might be interesting especially in extractions with complications. There are studies reporting late infections after the extraction of third molars, although they are usually related to lower third molars, which are more prone to this complication (13).

Pain seemed to lineally decrease and practically disappeared on day 4. Other studies about lower third molar extraction showed that pain is more persistent, and disappears around day 6 or 7 (11). Despite the fact that the VAS score for pain across the 7 days showed a progressive reduction, the outliers on day 6 and day 7 might be caused by the anxiety of return to the hospital for suture removal (14).

It is interesting to compare the reported pain scores with Colorado-Bonnin *et al.* and Sancho-Puchades *et al.*, (1,2) considering that extractions were performed at the same institution, with the same surgical technique and under local anesthesia. However, in the first report only one lower third molar was extracted per patient and in the second paper the 4 wisdom teeth were removed under conscious sedation. In comparison, pain scores observed in the present study were slightly lower on days 4 to 7, although during the first three days they were similar.

Unlike lower third molar extractions, which are often removed for pain or swelling, in the present study only 50% of upper third molars had previous symptoms (1). The present study seems to contradict other reports on third molars, since previous symptoms did not seem to predict a delayed recovery (15).

No significant difference were found regarding gender and pain. Although a previous paper (1) on QoL after lower third molar extraction reported more pain in women, and other authors (16,17) have mentioned that this symptom seems to last longer in females, our results showed quite similar pain outcomes in men and women.

It was not possible to analyze pain scores based on the experience of the professional considering that 85% of the operations were conducted by first-year postgraduate students. This factor might influence the results since some studies have shown that experienced surgeons seem to have less complications (3,18).

Therefore the major complaints referred by patients were inflammation and discomfort related to the chewing ability (10% to 44%). As a result, the quality of life was basically affected by eating difficulties. These figures were considerably higher after removing the 4 third molars in the same appointment (57.1%) (2).

Changes in chewing ability and trismus, were reported by 7% and 33%, respectively. However, after lower third molar removal up to 80% of patients report changes in chewing ability (1). When 4 third molars are extracted under conscious sedation, half of the patients report trismus. Therefore, for upper third molar extraction there is less chance of developing trismus or masticatory prob-lems, probably related to a shorter operating time and lesser involvement of elevators of the mandible, such as masseters and pterygoids (19).

Around 3 % of patients noticed changes in their voice and their ability to speak. There is a great difference when the 4 third mo-lars are extracted in the same operation, with 10% noticing voice changes and a 60% experiencing difficulties to be understood by other people. (2) In the extraction of mandibular third molars 20% perceived changes in their voice, while 57 % had problems to be understood. Therefore, extraction of upper molars had less impact on these abilities than extraction of lower third molars. Indeed this minor impairment to speak might be related to the lower prevalence of trismus. This complication might be specially relevant in some professions that require to speak like teachers, waiters among others.

Around one fourth of patients showed an alteration in taste perception for the first 7 days. Similar results were found in the study of Colorado Bonnin *et al.* (1) (15% quite a lot and 27.5 % a little). In the case of lower molars this could be related with lingual nerve function or use of chlorhexidine mouthrinses. Indeed, in upper molar extractions rinses with chlorhexidine digluconate might play an important role in taste perception, especially increasing the threshold for salty taste (1,4,12).

Only 5 % of the patients discontinued their work, much less than in lower third molar removal (50.5%) (1). However, 47% of patients were accompanied, similar to lower third molar removal, and in both cases most accompanying persons discontinued their work.

The McGrath *et al.* cohort study confirms that after third molar extraction there is a deterioration in the quality of life in the short term, but better oral health in the long term, especially observed in cases of previous pericoronitis (10).

Regarding the level of satisfaction, 93 % of patients were satisfied and believed that their problem was solved. Most studies show similar satisfaction rates above 90 % (1-3,5,9).

A variable that we did not take into account is tobacco use after surgery, which could affect postoperative pain levels, especially in extractions that require more aggressive surgical techniques or are more prone to postoperative infection.

The analysis of the repercussions of the extraction on patients’ QoL is important for an optimal preoperative assessment and development of appropriate indications after the surgery. Furthermore, it enables the surgeon to give the patient realistic information on the postoperative course, and helps the patient to choose the best moment to undergo the procedure, thus minimizing major interferences with everyday life.

In conclusion, most patients were satisfied with the treatment and believed that their problem was solved although there was a small percentage of patients that would not repeat it nor recommended it. Tuberosity fracture significantly increased the postoperative pain. The patients’ major complaints were inflammation and chewing difficulties. Upper third molar extraction had a small negative impact on quality of life, considering that most of patients continued their normal activities.
